# Real-Life Implementation of a GPS-Based Path-Following System for an Autonomous Vehicle

**DOI:** 10.3390/s18113940

**Published:** 2018-11-14

**Authors:** Alexander de Winter, Simone Baldi

**Affiliations:** Delft Center for Systems and Control, Delft University of Technology, Mekelweg 2, 2624 CD Delft, The Netherlands; ajdewinter@live.nl

**Keywords:** autonomous driving, path-follower, Spatial Dual GPS, real-life tests

## Abstract

This work is meant to report on activities at TU Delft on the design and implementation of a path-following system for an autonomous Toyota Prius. The design encompasses: finding the vehicle parameters for the actual vehicle to be used for control design; lateral and longitudinal controllers for steering and acceleration, respectively. The implementation covers the real-time aspects via LabVIEW from National Instruments and the real-life tests. The deployment of the system was enabled by a Spatial Dual Global Positioning System (GPS) system providing more accuracy than the regular GPS. The results discussed in this work represent the first autonomous tests on the Toyota Prius at TU Delft, and we expect the proposed system to be a benchmark against which to test more advanced solutions. The tests show that the system is able to perform in real-time while satisfying comfort and trajectory tracking requirements: in particular, the tracking error was within 16 cm, which is compatible with the 13 cm precision of the Spatial Dual GPS, whereas the longitudinal and lateral acceleration are within comfort levels as defined by available experimental studies.

## 1. Introduction

The last decades have seen an increasing amount of research on automated driving. What started as driving assist systems, such as cruise control or a lane departure warning system, expanded rapidly into advanced driving assist systems (ADAS) that are capable of stopping the vehicle in critical situations, keeping the vehicle in its own lane and regulating the vehicle velocity from 0 to 130 km/h with adaptive cruise control (cf. [1,2,3] among other references).

This work is meant to report on some activities at TU Delft on the design and implementation of a path-following system for an autonomous Toyota Prius: the deployment of the system was enabled by a Spatial Dual GPS system providing more accuracy than the regular GPS. The focus of this work is on the path-follower system: such system receives a trajectory (in terms of desired position and velocity) from a path planner. While extremely relevant, higher-level tasks such as changing the trajectory when the surroundings change (e.g., other vehicles merging in front of the autonomous vehicle, pedestrians crossing the road) are not addressed here. The design encompasses: finding the vehicle parameters for the actual vehicle to be used for control design; lateral controller for steering and longitudinal controller for acceleration. The implementation covers the real-time aspects via LabVIEW from National Instruments and the real-life tests performed at the Valkenburg Naval Air Base. While the goal of the control would be to make the steering and acceleration as human-like as possible, achieving such task would require very advanced strategies such as non-linear model predictive controllers (cf. [4,5,6,7] and references therein). Such strategies are well known for being computationally intensive and not straightforward to deploy (due to many design parameters such as control horizon, prediction horizon, terminal constraints, multi-objective weights, etc.). Therefore, it is worth remarking that the controllers presented here have been designed with the aim of real implementation, with few parameters to be tuned and low requirements from the computational point of view. The focus was on relatively simple solutions rather than on optimality.

The well-known book “Vehicle Dynamics and Control” [8] refers to several control algorithms for path following, all of them using to some extent the information on the curvature of the desired path: the most basic algorithms use the information of the path at the current position [9,10,11], while more advanced algorithms use a look-ahead distance and calculate the desired steering angle with use of some future location of the vehicle [12,13]. The controller selected for implementation in this work is the Future Predictive Controller of [14]. Even though more advanced strategies exist, e.g., based on optimality arguments [15,16], we found that the strategy in [14] has few design parameters and allows for fast implementation. For the longitudinal acceleration, we used a proportional-derivative strategy, which is standard even in the more advanced strategies for longitudinal control [1,2,3].

The system is developed in LabVIEW from National Instruments [17]. LabVIEW gives the opportunity to have real-time simulations, and most importantly, it is compatible with the National Instruments hardware that the Toyota Prius is equipped with. A code programmed in LabVIEW can be compiled to a National Instruments device from which it is possible to run the code in real-life systems [18]. The real-life tests show that the system is able to perform in real-time while satisfying comfort and trajectory tracking requirements: in particular, the tracking error was within 15.8 cm (compatible with the Spatial Dual GPS precision, which is of around 13 cm according to the manufacturer) and the lateral/longitudinal acceleration were within comfort levels as defined in well-known empirical tables [19,20]. As the results discussed in this work represent the first autonomous tests on the Toyota Prius at TU Delft, we expect the proposed system to be a benchmark against which to test more advanced solutions in the future.

This paper is organized as follows. Section 2 introduces the vehicle we consider. Section 3 outlines the control design for both lateral and longitudinal dynamics. Section 4 discusses the LabVIEW implementation and validates the model with real-time simulations. Section 5 deals with the real-life experiments and conclusions are drawn in Section 6. Comfort levels of lateral/longitudinal acceleration are briefly recalled in the Appendix.

## 2. The Vehicle

**Figure 1:**
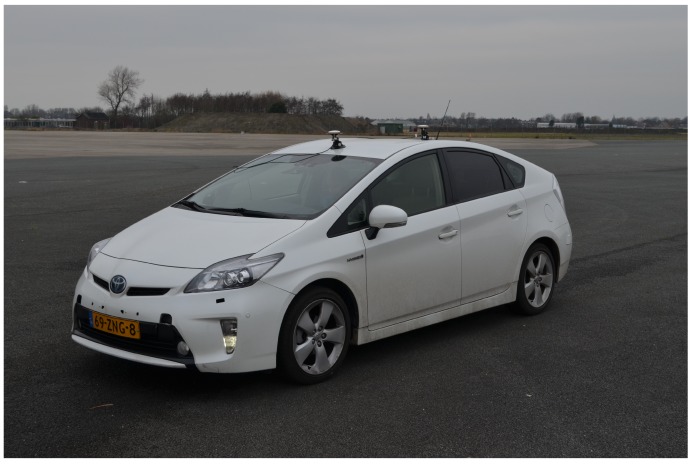
The Toyota Prius used for this work (the two devices on the roof of the vehicle are part of the Spatial Dual GPS).

**Figure 2:**
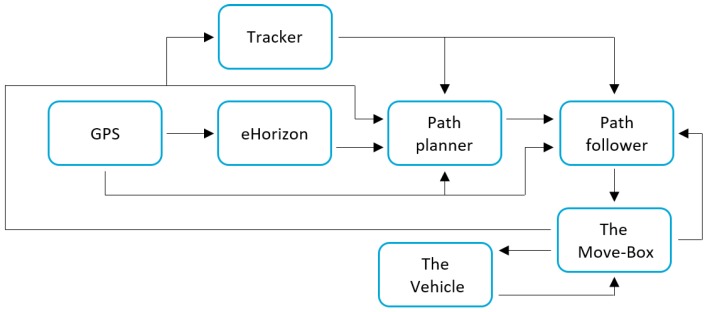
Schematic view of the autonomous vehicle system.

Within the Dutch Automated Vehicle Initiative (DAVI), TU Delft and other partners are working on the development of high automated vehicles for research and demonstrations on public roads. Among other vehicles, a Toyota Prius is being equipped to such purposes (cf. Figure 1), e.g., with sensors and radar to achieve 360 degrees of sensing. The autonomous driving system in the Prius encompasses a set of modules, as shown in Figure 2: some of the systems are ready to use, whereas others are still under development. Most important to the scope of this work is that the vehicle has been equipped with a system that is capable of controlling the steering, acceleration and brakes of the vehicle. This system is called Move-Box, developed by TNO (Dutch for: Netherlands Organisation for Applied Scientific Research), and serves also as an interface between the PCI eXtensions for Instrumentation (PXI) mounted on the vehicle. An eHorizon system by Continental [21] is capable of placing the vehicle inside a high-definition map with use of GPS. For higher level tasks (not considered in this work), such as path planning tasks, the vehicle is able to verify the GPS location with use of camera images: furthermore, the tracker can recognize objects and track them via the radars and cameras that are mounted on the vehicle.

The focus of this work is on the path-follower system: such systems receives a trajectory (in terms of desired position and velocity) from the path planner that the autonomous vehicle must follow. Tasks such as changing the trajectory when the surroundings change (e.g., other vehicles merging in front of the autonomous vehicle, pedestrians crossing the road) are decided at the tracker/path planner level, and will not be addressed here. From the sensing point of view, it is worth mentioning that the GPS installed on the Toyota Prius is an advanced system from a company named Advanced Navigation. The GPS system is called Spatial Dual and uses two antenna mounted on the roof of the vehicle as shown in Figure 1. Basically, Spatial Dual is a ruggedized miniature GPS which is augmented with sensor fusion from an inertial navigation system (INS) and attitude and heading reference system (AHRS). This makes it more precise than regular GPS systems with respect to position, velocity, acceleration and orientation.

The model of the vehicle used for control design is the standard bicycle model [22,23,24], which can be summarized as follows

(1) $$\left[\begin{array}{c} {\dot{v}}_{y}\\ \dot{r} \end{array}\right]=\left[\begin{array}{cc} -\frac{C_{\alpha f}+C_{\alpha_{r}}}{mv_{x}} & -v_{x}+\frac{l_{r}C_{\alpha_{r}}-l_{f}C_{\alpha_{f}}}{mv_{x}}\\ \frac{l_{r}C_{\alpha r}-l_{f}C_{\alpha_{f}}}{I_{z}v_{x}} & \frac{-l_{r}^{2}C_{\alpha_{r}}-l_{f}^{2}C_{\alpha_{f}}}{I_{z}v_{x}} \end{array}\right]\left[\begin{array}{c} v_{y}\\ r \end{array}\right]+\left[\begin{array}{c} \frac{C_{\alpha_{f}}}{m}\\ \frac{l_{f}C_{\alpha_{f}}}{I_{z}} \end{array}\right]\delta$$

where $v_{y}$ is the longitudinal velocity, *r* is the yaw rate, δ is the wheel steering angle, and the other parameters are defined in Table 1. Note that the longitudinal velocity $v_{x}$ is a time-varying parameter of the bicycle model. The steering ratio $k_{\delta }$ is needed to convert the wheel steering angle into the actual angle of the steering wheel given by the driver and constrained in $\left[-\delta_{max},\delta_{max}\right]$ (please note that we use the term “wheel steering angle” for the angle of the front wheels, and the term “angle of the steering wheel” for the steering angle of steering wheel, i.e., the rotation of the steering wheel). $\tau_{\delta }$ is the time constant of a first-order filter used to model the delay of the steering actuator. In Equation (1), the tire model is taken as a linear model, as typically assumed in the bicycle model: this implies that the model is valid as soon as the vehicle operates at a linear regime (low acceleration/deceleration and smooth cornering) [22,25]. The vehicle parameters in Table 1 were initially unknown and have been found as explained in the following subsection.

### Vehicle Parameters

To find the distance of the front and rear axles from the Centre of Gravity (CoG), it was first necessary to determine the location of the CoG. To locate its position, it was necessary to measure the weight that rests on the front axle and on the rear axle. Unfortunately, we could not rely on the vehicle manufacturer information, because all the equipment added to the vehicle had likely changed the position of the CoG. Therefore, weighting has been performed on a weightbridge with two persons in the front chairs.

The steering parameters $k_{\delta }$, $\delta_{max}$ and $\tau_{\delta }$ have been retrieved from the data-sheets of Toyota. Finally, the values of the cornering stiffness and the moment of inertia are found with use of data fitting. In particular, while driving the vehicle, the angle of the steering wheel, longitudinal velocity, lateral acceleration and yaw rate have been recorded. The last two quantities have been measured via the INS mounted of the vehicle. Then, the missing parameters have been fit on the recorded data with a non-linear least squares method by using the algorithm lsqnonlin in Matlab. The vehicle has been driven in such away to have the tires working in a linear regime [26,27], in line with the linear bicycle model in Equation (1). The experiments have been performed at the Valkenburg Naval Air Base, a location that provides the appropriate space for this type of tests.

**Figure 3:**
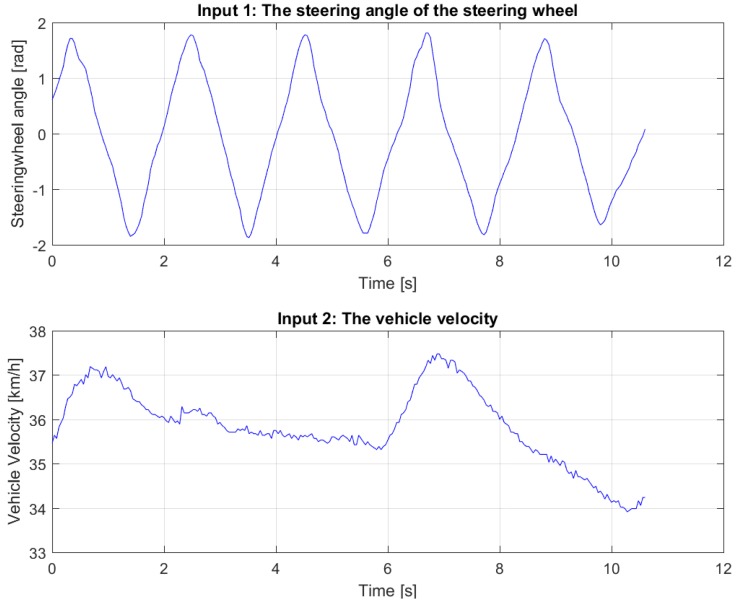
The inputs of one dataset.

**Figure 4:**
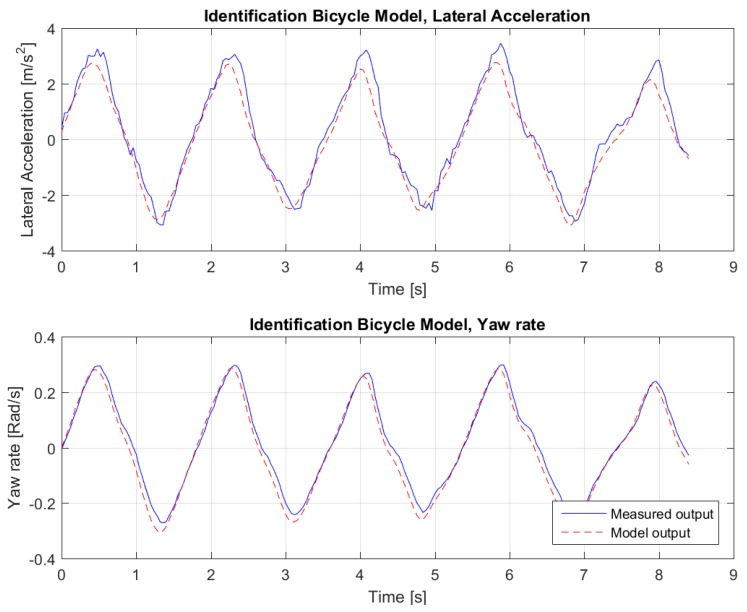
Data fit.

Several datasets have been used for identification and validation: one such set is shown in Figure 3. The result of the fitting for this dataset can be seen in Figure 4. As the Variance Accounted For (*VAF*)

(2) $$VAF=(1-\frac{variance(y-y_{est})}{variance(y)})\times 100$$

is 96.36% for the lateral acceleration and 98.41% for the yaw rate, we conclude that the resulting bicycle model can be used for control design purposes, as explained in the next section.

## 3. Controls

The goal of the controllers is to make the steering and acceleration as human-like as possible. While it is acknowledged that advanced non-linear model predictive control techniques would make this possible, a primary goal was to deliver a simpler solution which could be more easily designed, validated and tested. Most importantly, the controller should be able to run in real-time with low computational requirements.

### 3.1. Lateral Control

The controller selected among many possible options was the Future Predictive Controller [14]. This control algorithm makes use of a future location to generate the angle of the steering wheel. The controller can be written as follows

(3) $$\begin{array}{cc} \theta_{e} & =\theta -\theta_{p}\left(t\right)\\ f_{x} & =L_{f}\cos\left(\theta \right)+c_{x}\\ f_{y} & =L_{f}\sin\left(\theta \right)+c_{y}\\ y_{ef} & =-\left(f_{x}-p_{x}\right)\sin\left(\theta \right)+\left(f_{y}-p_{y}\right)\cos\left(\theta \right)\\ L_{f} & =k_{f}v_{x}\\ \delta & =k_{h}\sin\left(\theta_{e}\right)+\frac{k_{s}y_{ef}}{v_{x}} \end{array}$$

where the first equation represents the heading error, $\left(c_{x},c_{y}\right)$ is the current location of the vehicle, $\left(f_{x},f_{y}\right)$ is the future location of the vehicle, $y_{ef}$ is the future lateral error, and $L_{f}$ is the look-ahead distance dependent on $v_{x}$ [12].

**Figure 5:**
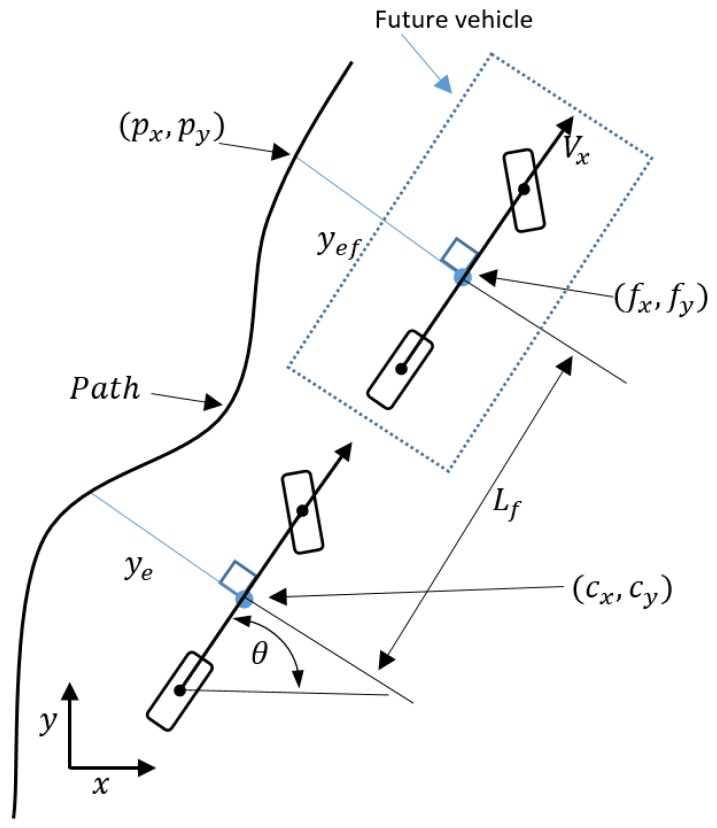
The future vehicle of the future prediction control algorithm.

A graphical explanation of the control strategy is illustrated in Figure 5. The tuning of this control algorithm is done with three different parameters, $k_{f}$, $k_{s}$ and $k_{h}$. The parameters have been tuned using the bicycle model, in such a way that the tracking error is small while the lateral acceleration is within human comfort limits. The optimization has been performed using patternsearch in Matlab. Clearly, the fact that only three parameters need to be tuned make the design and tuning quite straightforward, whereas the same might not be true for other control strategies like linear quadratic or model predictive control. The following rules of thumb can be derived: by increasing $k_{s}$ and $k_{h}$ the steering is more aggressive: the error will in general be smaller, but at the price of high lateral acceleration and sometimes instability. Increasing $k_{f}$ will result in a larger look-ahead distance: if the distance is too large the vehicle will cut corners, and if this distance is too small the vehicle will oscillate.

### 3.2. Longitudinal Control

The longitudinal control also satisfies the criteria of simplicity of design and tuning. It is a Proportional-Derivative (PD) action that regulates the acceleration of the vehicle [28]

(4) $$v_{e}\left(t\right)=v_{c}\left(t\right)-v_{p}\left(t\right)$$

(5) $$a_{c}\left(t\right)=K_{p}v_{e}\left(t\right)+K_{d}\frac{dv_{e}\left(t\right)}{dt}$$

where $v_{e}$ is the error in velocity, $v_{c}$ is the vehicles velocity, $v_{p}$ is the velocity that is requested by the path, and $a_{c}$ is controlled acceleration. The parameters of this control law are the proportional control gain $K_{p}$ and the derivative control gain $K_{d}$. These parameters have been tuned using the bicycle model, in such a way that the tracking error is small while the longitudinal acceleration is within human comfort limits. The comfort level for humans (both for longitudinal and lateral comfort) has been evaluated from experimental studies [19,20] that define threshold values of comfort: comfortable level, medium comfort level and discomfort level.

The tuning of $K_{p}$ and $K_{d}$ has been performed by trial and error: because in total we have only five parameters to tune, it is also possible to perform the tuning of the lateral and longitudinal controllers jointly via patternsearch, even though we have noticed minor differences as compared to a separated tuning. The resulting parameters are shown in Table 2, and their tuning has been performed by interfacing Matlab with LabVIEW as explained in the next section.

## 4. Real-Time Implementation

The program used to construct the system is LabVIEW from National Instruments. LabVIEW gives the opportunity to construct a Virtual Instrument (VI) that can be simulated when the time is synchronized to a timing source. This makes it possible to have real-time simulations. While such capabilities are also possible with other programs, e.g., Matlab, the main advantage of LabVIEW is its compatibility with the National Instruments hardware that the Toyota Prius is equipped with. This means that a code programmed in LabVIEW can easily be compiled to a National Instruments device from which it is possible to run the code in real-life systems.

For example, the Move-Box in the Toyota Prius (cf. Figure 2) is actuated by a PCI eXtension for Instrumentation (PXI) from National Instruments. The implementation of the system to the PXI can then be easily done, and then it is possible to test if the full system is able to run real-time. To generate a path the following conventions are adopted: the positions *x* and *y* are in a global coordinate system. The Universal Transverse Mercator coordinate system (UTM) is chosen. The heading angle is in radians, increasing counter clockwise from a polar axis that is drawn horizontal and pointed to the right. The velocity is in meters per second, even though in the plots it will be reported in km/h for better understanding. For the sake of readability, let us skip all the practical issues that must be taken into account when implementing a path follower, namely: the LLtoUTM submodule, which takes care of the conversion from latitude and longitude to UTM coordinates [29]; and the curve equation submodule, which finds the nearest path point and makes a curve fit to a part of the trajectory.

### Simulations

This section presents the simulation tests before going to the real-life tests. Some standard paths have been used for simulating and tuning the controllers. When looking for the best performance, the following cost functions have been considered:

- The Root Mean Square (RMS) of the error distance $y_{e}$
- The maximum value of $y_{e}$
- The minimum value of $y_{e}$
- The maximum lateral acceleration $a_{y}$

**Figure 6:**
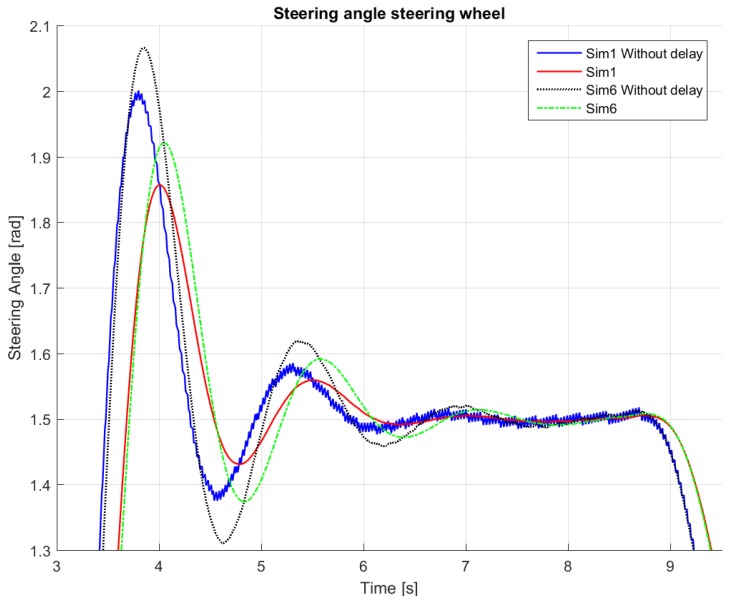
Angle of the steering wheel responses for evaluation of steering output.

Table 3, Table 4, Table 5 and Table 6 show how the performance changes after changing the different parameters for some representative values of $k_{f}$, $k_{s}$ and $k_{h}$ (the different simulations are numbered 1–8). The final settings used in the controller are the ones in Simulation 6 (and reported in Table 2). It has to be noted that, due to the noise in the GPS heading error, we found it beneficial to filter the heading error. The benefits of this action can be better seen from the smoother response in Figure 6, where the “delay” refers to a first-order lag element used to model the steering actuator. Note that, in Figure 6, the filter removes the fast oscillations present in the original control action.

While these simulations have been performed at 30 km/h, we also tried simulations at higher velocity (50 km/h): our findings were that the same control parameters did not work well at such velocity, and the hypothesis is that the control gains should be scheduled according to the velocity. In particular, we found that the steering response is highly dependent on the velocity of the vehicle.

## 5. Real-Life Experiments

**Figure 7:**
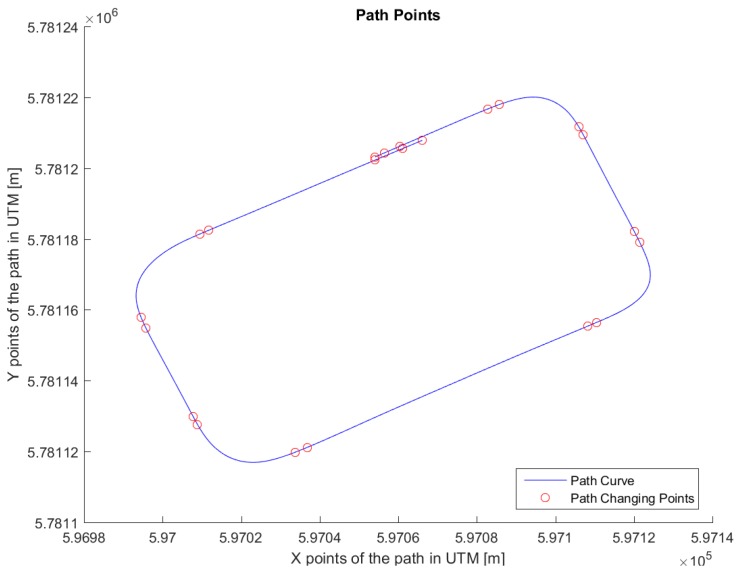
Trajectory for the test drive.

**Figure 8:**
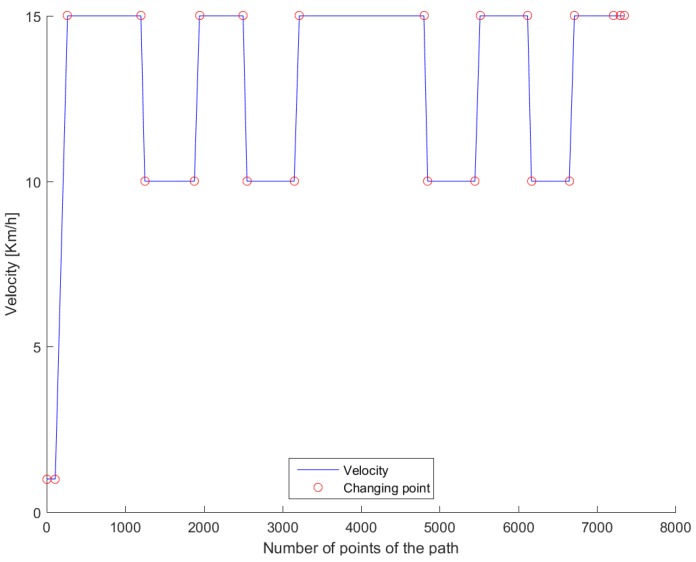
Velocity profile for the test drive.

The path in Figure 7 was used for real testing. The path was driven at Valkenburg Naval Air Base. Note that, because this path is driven with the actual vehicle, the end and beginning of the path do not coincide; in addition, the corners of the path are not identical and the lines between the corners are not completely straight. The velocity of the path is variable: this is achieved by imposing to the Move-Box appropriate acceleration and deceleration. The resulting velocities are approximately of 15 km/h at straight lines and 10 km/h at cornering. The velocity profile can be seen in Figure 8.

**Figure 9:**
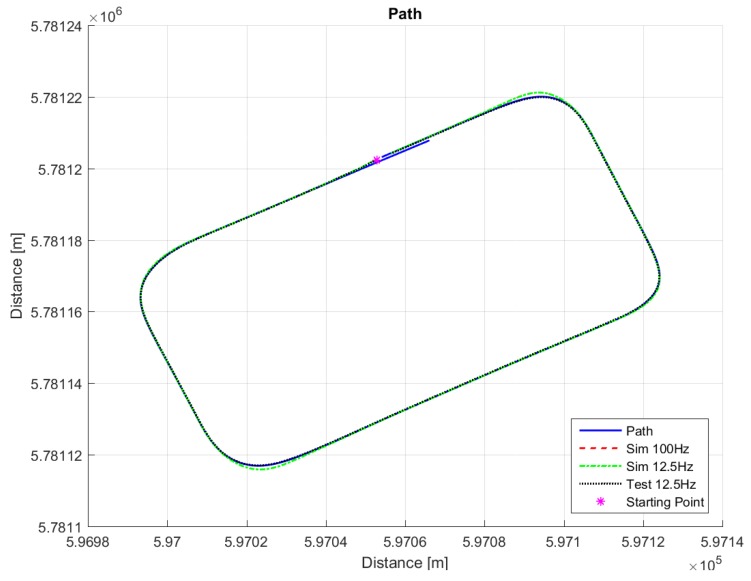
The path, simulation responses and the response from the test drive.

**Figure 10:**
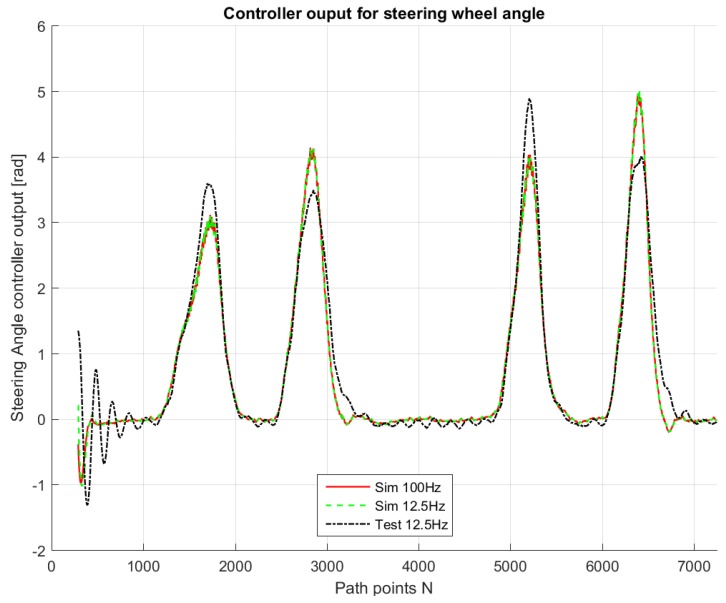
Controller output for simulations and test drive.

**Figure 11:**
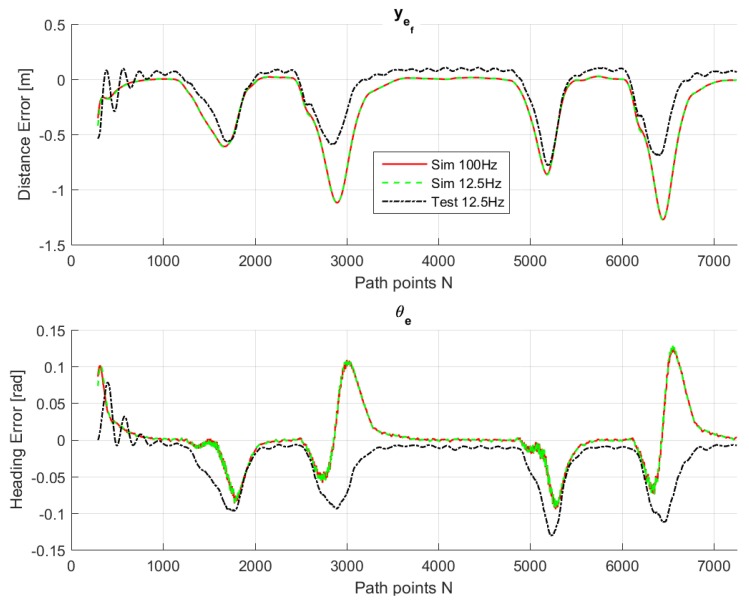
Lateral control inputs for simulations and test drive.

**Figure 12:**
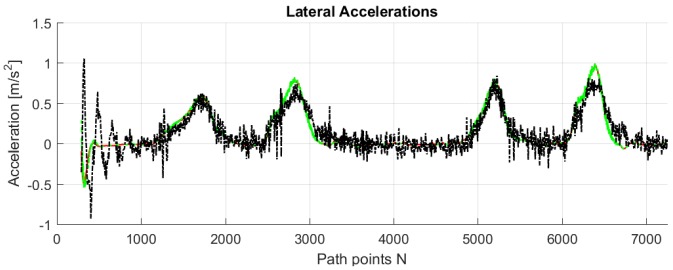
Lateral acceleration for simulations and test drive.

**Figure 13:**
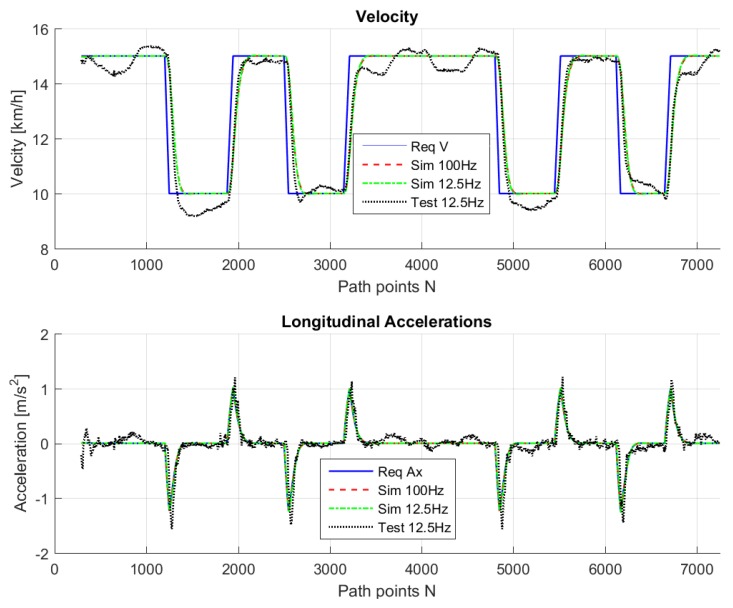
Longitudinal control output and resulting velocity for simulations and test drive.

For comparison purposes, we simulated the same path on the LabVIEW system, so that we are able to compare the real-life behavior with the simulated ones. Figure 9, Figure 10, Figure 11, Figure 12 and Figure 13 show that such behaviors are quite close. The actual distance error turns out to be smaller than in simulations, whereas the actual velocity tracking error turns out to be bigger. This is mainly due to actuator dynamics (power train dynamics) that cannot be modeled in the bicycle model.

The comparison between simulations and real-test shown in Table 7 reveal that the various errors are of a similar order of magnitude. It is worth mentioning that tests at different frequencies had to be performed, since the system on the actual vehicle had to work at 12.5 Hz. It can be noted that the maximum error is of around 16 cm, which is consistent with the accuracy of the Spatial Dual GPS on the Toyota Prius which is 13 cm. The minimum error is of the order of 1 m, which is because the vehicle smooths the corners. In addition, the accelerations are always within the expected comfort limits. For completeness, the comfort limits are reported in the Appendix.

### Supplementary Materials

Videos of other actual tests can be found at [30,31]. The two tests are performed at a maximum velocity of 20 km/h and 30 km/h, respectively. In these tests the velocities are higher than the 15 km/h reported in the previous section. These tests are meant to show the effectiveness of the path-following even at higher speeds. From the videos, it is possible to see the prediction used to generate the angle of the steering wheel. The steering wheel moves quite smoothly, in a human-like manner. In addition, it can be seen that the current velocity smoothly follows the desired velocity (which is pre-selected by the path planner), and, furthermore, no harsh acceleration is registered. As these results represent the first autonomous tests on the Toyota Prius at TU Delft, we expect the proposed system to be a benchmark against which to test more advanced solutions in the future.

## 6. Conclusions and Future Work

This paper reports some recent activities at TU Delft on the design and implementation of a path-following system for an autonomous Toyota Prius. The design encompassed: finding the vehicle parameters for the actual vehicle to be used for control design, and lateral controller for steering and longitudinal controller for acceleration. The implementation covered the real-time aspects via LabVIEW from National Instruments and the real-life tests. It is worth remarking that the controllers have been designed with the aim of straightforward implementation, with low requirements from the computational point of view. Therefore, the focus was on relatively simple solutions rather than on optimality. As the results discussed in this work represent the first autonomous tests on the Toyota Prius at TU Delft, we expect the proposed system to be a benchmark against which to test more advanced solutions. Future work will cover: a more advanced vehicle model (which requires measuring more parameters of the vehicle, especially during nonlinear regime); more advanced controllers (compatibly with the computational requirements of the equipment); and further development of all other autonomous driving modules. Finally, before it will be possible to do more research on vehicle control, especially during extreme manoeuvres, it will be required to have a better understanding of the Move-Box system.

## Figures and Tables

**Table 1:** Acquired vehicle parameters.

Parameter	Value	Symbol	Unit
Length from front axle to CoG	1.0868	$l_{f}$	m
Length from rear axle to CoG	1.6132	$l_{r}$	m
Vehicle mass	1590	*m*	kg
Cornering stiffness of the front wheels	22,200	$C_{\alpha_{f}}$	N/rad
Cornering stiffness of the rear wheels	22,200	$C_{\alpha_{r}}$	N/rad
Moment of inertia around Z axis	800	$I_{z}$	kg/m^2^
Steering ratio	14.6	$k_{\delta }$	-
Maximum angle of the steering wheel	7.592	$\delta_{max}$	rad
Dynamic steering time constant	0.2	$\tau_{\delta }$	s

**Table 2:** Control parameters.

	Value
$k_{f}$	1.1
$k_{s}$	0.7
$k_{h}$	1.0
$K_{p}$	0.3
$K_{d}$	1.18

**Table 3:** Evaluation parameters for variation $k_{s}$.

		Simulation 1	Simulation 2	Simulation 3
Steering Parameter	$k_{s}$	0.7	0.1	1.3
RMS	(m)	0.052	2.044	0.331
Max $y_{e}$	(m)	0.097	0.010	0.601
Min $y_{e}$	(m)	−0.099	−3.220	−0.001
Max $a_{y}$	(m/s^2^)	2.282	2.165	2.188

**Table 4:** Evaluation parameters for variation $k_{f}$.

		Simulation 1	Simulation 4	Simulation 5
Look-Ahead Parameter	$k_{f}$	1.1	0.1	2.1
RMS	(m)	0.052	0.769	1.633
Max $y_{e}$	(m)	0.097	0.002	2.708
Min $y_{e}$	(m)	−0.099	−1.184	−0.0001
Max $a_{y}$	(m/s^2^)	2.282	2.623	3.624

**Table 5:** Evaluation parameters for evaluation of steering output.

		Simulation 1	Simulation 6
RMS	(m)	0.052	0.052
Max $y_{e}$	(m)	0.097	0.104
Min $y_{e}$	(m)	−0.099	−0.100
Max $a_{y}$	(m/s^2^)	2.282	2.357

**Table 6:** Evaluation parameters for variation $L_{f}$.

		Simulation 7	Simulation 8
Feed-forward length $L_{f}$	(m)	9.2	12.2
RMS	(m)	0.393	0.285
Max $y_{e}$	(m)	0.209	0.559
Min $y_{e}$	(m)	−0.797	−0.366
Max $a_{y}$	(m/s^2^)	2.6566	2.5432

**Table 7:** Evaluation parameters for difference between simulation and real test.

Test		Simulation 100 Hz	Simulation 12.5 Hz	Real Test 12.5 Hz
RMS	(m)	0.332	0.333	0.122
Max $y_{e}$	(m)	0.113	0.114	0.158
Min $y_{e}$	(m)	−1.193	−1.195	−0.654
Max $a_{y}$	(m/s^2^)	0.981	1.014	1.063

**Figure A1:**
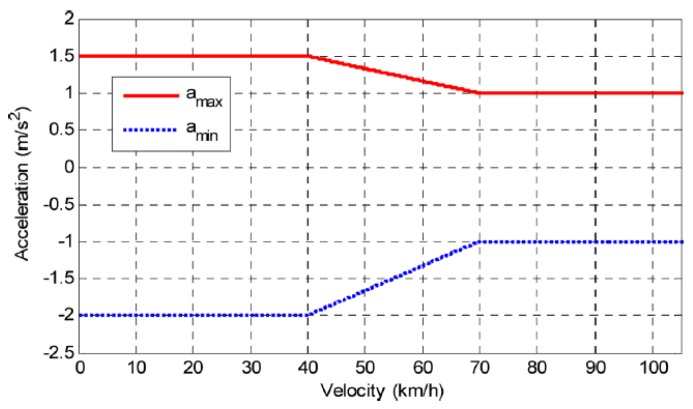
Velocity-dependent range of longitudinal accelerations.

## Appendix A. Acceleration for Human Comfort

The comfort level for humans in terms of lateral acceleration have been quantified in an experimental study [20]. Different levels have been found, as shown in Table A1.

**Table A1:** Lateral acceleration thresholds of comfort.

Lateral Acceleration (m/s^2^)	Consequence
0 $<a_{y}$ ≤ 1.8	Comfort level
1.8 $<a_{y}$ ≤ 3.6	Medium comfort level
3.6 $<a_{y}$ ≤ 5	Discomfort level
5 $<a_{y}$	Uncomfortable

The maximum and minimum longitudinal accelerations depend on the vehicle’s velocity and have been determined with use of human driving data [19]. These acceleration values are shown in Figure A1.
